# Irreversibility and Action of the Heat Conduction Process

**DOI:** 10.3390/e20030206

**Published:** 2018-03-20

**Authors:** Yu-Chao Hua, Tiao Zhao, Zeng-Yuan Guo

**Affiliations:** Key Laboratory for Thermal Science and Power Engineering of Ministry of Education, Department of Engineering Mechanics, Tsinghua University, Beijing 100084, China

**Keywords:** irreversibility, heat conduction, Lyapunov function, least action principle, entransy, entropy

## Abstract

Irreversibility (that is, the “one-sidedness” of time) of a physical process can be characterized by using Lyapunov functions in the modern theory of stability. In this theoretical framework, entropy and its production rate have been generally regarded as Lyapunov functions in order to measure the irreversibility of various physical processes. In fact, the Lyapunov function is not always unique. In the represent work, a rigorous proof is given that the entransy and its dissipation rate can also serve as Lyapunov functions associated with the irreversibility of the heat conduction process without the conversion between heat and work. In addition, the variation of the entransy dissipation rate can lead to Fourier’s heat conduction law, while the entropy production rate cannot. This shows that the entransy dissipation rate, rather than the entropy production rate, is the unique action for the heat conduction process, and can be used to establish the finite element method for the approximate solution of heat conduction problems and the optimization of heat transfer processes.

## 1. Introduction

A physical process is time-reversible if the dynamics of the process remain unchanged when the sequence of time is reversed, indicating that the direction of time does not matter for a reversible process. Actually, the direction of time is essentially important in practice, since most practical processes obviously display the “one-sidedness” of time, i.e., irreversibility [1,2,3,4,5,6,7]. For example, when some part of a solid is heated and then the solid is isolated from the surroundings, we observe that its temperature gradually becomes uniform, and a reversed process will not happen in reality. The second law of thermodynamics has been used to characterize the phenomenon of irreversibility [8], with entropy, as proposed by Clausius [9], as the central concept. The second law indicates that the total entropy of an isolated system continues increase until reaching its maximum value at the equilibrium state. This conclusion can be extended to systems involving heat and mass exchange with outside systems.

The entropy variation, δS, is made of two distinct parts,

(1) $$\delta S=\delta S_{e}+\delta S_{i}$$

where $\delta S_{e}$ is the entropy flow (that is, the transfer of entropy across the boundaries), and $\delta S_{i}$ is the entropy production within the system. It should be emphasized that only irreversibility can lead to entropy production [1]. Irreversible processes involve the one-sidedness of time, and the positive time direction is associated with the increase of entropy [6,7]. Additionally, in the view of the stability theory [10,11], entropy variation can be regarded as a Lyapunov function of an isolated system [12]. Generally, Lyapunov functions are scalar functions that can be used to prove the stability of an equilibrium state of a dynamic system. If a system holds a positive-definite (or negative-definite) function, and its time derivative function has the opposite sign, the system is stable, and this function is a Lyapunov function of the system [10]. Moreover, the Lyapunov function should reach its extremum at the stable state of the system, and this stable state can be called the attractor. For an isolated system shifting from a non-equilibrium state to the equilibrium state, the equilibrium state is the attractor and the entropy variation serves as a Lyapunov function, since it has been proven that [12]

(2) $$\begin{array}{l} \delta S=\delta S_{i}>0\\ {\delta S|}_{\mathrm{eq}}=0\\ {\frac{\partial \delta S}{\partial t}|}_{\mathrm{eq}}<0 \end{array}$$

In this case, irreversibility actually means that a Lyapunov function exists and the equilibrium state is the attractor of non-equilibrium states [12]. Furthermore, the theoretical framework of Lyapunov functions can be used to handle the irreversibility problems in a transport process from an unsteady state to the steady state. Prigogine proposed that the steady state of a linear irreversible process is characterized by a minimum value of entropy production rate, which has been called minimum entropy production principle [1,12,13,14]. This principle implies that for a linear transport process, the entropy production rate is a Lyapunov function and the steady state is its attractor [1,14].

As stated above, the existence of Lyapunov functions reflects irreversibility, and entropy and its production rate have been generally regarded as Lyapunov functions for various irreversible physical processes. However, in stability theory, the Lyapunov function is not always unique [10], indicating that for a specific irreversible process, entropy and its production rate may not be the only choice to tackle the irreversibility problem. For example, entransy [15,16] and its dissipation rate can also serve as Lyapunov functions for heat conduction processes in order to reflect the processes’ irreversibility. The emergence of entransy theory is due to the deficiency of the entropy production rate in handling heat conduction problems [17,18,19,20]. Although the entransy theory has been proven to be efficient for handling the heat transfer problems [16], a rigorous proof is still needed to clarify that the entransy and its dissipation rate are indeed associated with the irreversibility of the heat conduction process. This could provide a more solid physical basis of the entransy theory.

## 2. Irreversibility of the Heat Conduction Process

In the analogy between heat conduction and electrical conduction, Guo et al. [15] proposed a new quantity, entransy, corresponding the electrical potential energy,

(3) $$G=\frac{UT}{2}=\frac{C_{V}T^{2}}{2}>0$$

in which *U* is internal energy and *C_V_* is volumetric heat capacity. The entransy is always positive and characterizes the capability for thermal transport. Its dissipation rate is the dot product of heat flux and minus temperature gradient [15],

(4) $$\sigma_{g}=-q\cdot \nabla T$$

The combination of the above equation and Fourier’s heat conduction law, q=−k∇T, yields,

(5) $$\sigma_{g}=k{\left(\nabla T\right)}^{2}$$

What follows is a rigorous proof to demonstrate that entransy and its dissipation rate can serve as the Lyapunov functions to reflect the irreversibility of the heat conduction process. Basically, irreversibility can emerge in two situations [12]: (1) an isolated system from a non-equilibrium state to the equilibrium state; (2) a non-equilibrium system from an unsteady state to the steady state.

### 2.1. Irreversibility of Isolated System from Non-Equilibrium State to Equilibrium State

**Figure 1:**
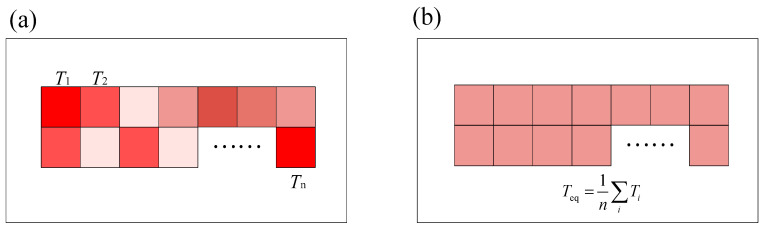
An isolated system involving the heat conduction process only: (**a**) The system consists of n solid blocks with various temperatures at the beginning; (**b**) The system reaches the equilibrium state with a uniform temperature distribution.

Figure 1 shows an isolated system involving only the heat conduction process. It consists of n solid blocks with various temperatures (*T*_1_, *T*_2_, ⋯, *T_n_*) at the beginning (as shown in Figure 1a). Then, the isolated system will be driven from the non-equilibrium state to the equilibrium state by the heat conduction between those solid blocks. During this process, the temperature within this system will gradually become uniform (as shown in Figure 1b). In the initial non-equilibrium state, the total entransy of the system is given by

(6) $$G_{0}=\sum_{i}^{n}\frac{U_{i}T_{i}}{2}=\sum_{i}^{n}\frac{C_{V}T_{i}^{2}}{2}$$

in which *U_i_* is the internal energy of block *i*, and $U_{i}=C_{V}T_{i}$. When the system is in equilibrium, the total entransy becomes,

(7) $$G_{\mathrm{eq}}=n\frac{C_{V}T_{e}^{2}}{2}$$

with the equilibrium temperature calculated via the energy conservation law,

(8) $$T_{\mathrm{eq}}=\frac{\sum_{i}^{n}T_{i}}{n}$$

The difference between *G*_eq_ and *G*_0_ is given by,

(9) $$\begin{array}{ll} G_{\mathrm{eq}}-G_{0} & =\frac{C_{V}{\left(\sum_{i}^{n}T_{i}\right)}^{2}}{2n}-\sum_{i}^{n}\frac{C_{V}T_{i}^{2}}{2}\\ & =\frac{C_{V}}{2}\left\{\frac{1}{n}{\left(\sum_{i}^{n}T_{i}\right)}^{2}-\sum_{i}^{n}T_{i}^{2}\right\} \end{array}$$

According to the Chebyshev’s sum inequality [21],

(10) $$\frac{1}{n}\sum_{i}^{n}T_{i}^{2}\geq {\left(\frac{1}{n}\sum_{i}^{n}T_{i}\right)}^{2}$$

we have,

(11) $$G_{\mathrm{eq}}-G_{0}=\delta G\leq 0$$

which indicates that the total entransy of the isolated system will decrease in the process from the non-equilibrium state to the equilibrium state; that is to say, the first-order derivative of the total entransy is negative. Additionally, we need to further clarify the second-order variation of the total entransy when the equilibrium state has been achieved, with ${\delta G|}_{\mathrm{eq}}=0$. In this case, the second-order variation of the total entransy is given by

(12) $${\delta^{2}G|}_{\mathrm{eq}}=\delta^{2}\left[n\frac{C_{V}T_{\mathrm{eq}}^{2}}{2}\right]=nC_{V}{\left(\delta T_{\mathrm{eq}}\right)}^{2}=nC_{V}{\left(\frac{1}{n}\sum_{i}^{n}\delta T_{i}\right)}^{2}>0$$

Since the heat capacity, *C_V_*, should be positive, the second-order variation of the total entransy is positive.

Therefore, according to the deviation above, we have,

(13) $$\min\left\{G\right\}\Leftrightarrow \{\begin{array}{c} G>0\\ \delta G<0\\ {\delta G|}_{\mathrm{eq}}=0\\ {\delta^{2}G|}_{\mathrm{eq}}>0 \end{array}$$

which indicates that the total entransy is at minimum for an isolated system in the equilibrium state achieved merely by the heat conduction process. Moreover, in the theoretical framework of the Lyapunov stability theory, for the process whereby an isolated system shifts from a non-equilibrium state to the equilibrium state, the final equilibrium state is the attractor, and the variation of the total entransy, δG, can serve as a Lyapunov function, since δG≤0 and $\delta^{2}G$. This indicates that the equilibrium state is stable and the entransy change reflects the irreversibility of the process when an isolated system shifts from a non-equilibrium state to the equilibrium state driven only by the heat conduction process.

### 2.2. Irreversibility of a Non-Equilibrium System Shifting from an Unsteady State to the Steady State

**Figure 2:**
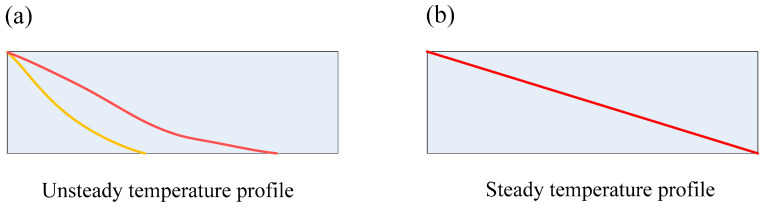
A non-equilibrium system from the unsteady state to the steady state: (**a**) an unsteady state; (**b**) the steady state.

Here, we turn to the case of a non-equilibrium system. As shown in Figure 2, as the initial condition, the system has a uniform temperature of *T*_0_ at *t* = 0; after *t* > 0, both the left and right sides are in contact with heat sinks of temperature *T_h_* and *T*_0_ (*T_h_* > *T*_0_), respectively, while the lateral boundaries are kept adiabatic. The boundary conditions are time-independent in the example. In this case, since the temperature difference always exists as *t* > 0, the system is non-equilibrium; it will undergo a process from an unsteady state (as shown in Figure 2a) to the steady state (as shown in Figure 2b).

Referring to Equations (4) and (5), the total entransy dissipation rate over the volume, *V*, is given by,

(14) $$\sigma_{G}=\int_{V}\sigma_{g}dV=\int_{V}k{\left(\nabla T\right)}^{2}dV>0$$

which is definitely positive. Therefore, once the time derivative of the total entransy dissipation rate is proven to be definitely negative, $\partial \sigma_{G}/\partial t<0$, the total entransy dissipation rate can serve as a Lyapunov function for a non-equilibrium system shifting from an unsteady state to the steady state. In this case, the time derivative of the total entransy dissipation rate is given by,

(15) $$\begin{array}{cc} \frac{\partial \sigma_{G}}{\partial t} & =\frac{\partial }{\partial t}\int_{V}k\nabla T\cdot \nabla TdV\\ & =\int_{V}2k\nabla T\cdot \nabla \frac{\partial T}{\partial t}dV\\ & =-\int_{V}2k\frac{\partial T}{\partial t}\nabla^{2}TdV+\int_{V}2k\nabla \cdot \left(\frac{\partial T}{\partial t}\nabla T\right)dV \end{array}$$

Using the energy conservation equation,

(16) $$C_{V}\frac{\partial T}{\partial t}=k\nabla^{2}T$$

and Stokes’ theorem, we have,

(17) $$-{\int_{V}2kC_{V}\left(\frac{\partial T}{\partial t}\right)}^{2}dV+\int_{V}2k\nabla \cdot \left(\frac{\partial T}{\partial t}\nabla T\right)dV=-{\int_{V}2kC_{V}\left(\frac{\partial T}{\partial t}\right)}^{2}dV+\int_{\partial V}2k\left(\frac{\partial T}{\partial t}\nabla T\right)dS$$

When the boundary conditions are time-independent, Equation (17) becomes

(18) $$-{\int_{V}2kC_{V}\left(\frac{\partial T}{\partial t}\right)}^{2}dV+\int_{\partial V}2k\left(\frac{\partial T}{\partial t}\nabla T\right)dS=-{\int_{V}2kC_{V}\left(\frac{\partial T}{\partial t}\right)}^{2}dV<0\Rightarrow \frac{\partial \sigma_{G}}{\partial t}<0$$

which indicates the time derivative of the total entransy dissipation rate is definitely negative. Moreover, as the system reaches the steady state, ∂T/∂t=0, Equation (18) can lead to

(19) $$\frac{\partial T}{\partial t}=0\Rightarrow -{\int_{V}2kC_{V}\left(\frac{\partial T}{\partial t}\right)}^{2}=0\Rightarrow \frac{\partial \sigma_{G}}{\partial t}=0$$

Equation (19) implies that the total entransy dissipation rate can be at its minimum in the steady state. Therefore, the final steady state is the attractor, and the total entransy dissipation rate serves as a Lyapunov function with $\sigma_{G}>0$ and $\partial \sigma_{G}/\partial t<0$ to characterize the irreversibility of the heat conduction process for a non-equilibrium system shifting from an unsteady state to the steady state.

## 3. Principle of Least Action for Heat Conduction Process

The principle of least action can provide some insights into physical phenomena, and it can be very useful in engineering applications [22,23,24]. For the heat conduction process, the least action principle can be used to derive Fourier’s heat conduction law, and then to optimize heat transfer problems. Several works [17,25,26] related to the least action principles of the heat conduction process have been published. Here, we will give a brief review of this topic to highlight that the action of the heat conduction process is unique, which is associated with the entransy dissipation rate, rather than the entropy production rate.

### 3.1. Deriving Fourier’s Law from Entransy Dissipation Rate

In a previously published work [25], we examined the actions of generalized linear transport processes, including heat conduction and mass diffusion, etc.; it was concluded that the dot product of generalized flux and generalized force in the phenomenological law is the action, the variation of which provides the corresponding constitutive relation. Particularly for the heat conduction process, it is the entransy dissipation rate, rather than the entropy production rate, that can be used to derive the principle of least action that corresponds to Fourier’s heat conduction law.

We begin by deriving the constitutive relation on the basis of the entropy production rate. In this case, the entropy production rate is written as [8]

(20) $$\sigma_{S}=\frac{-q\cdot \nabla T}{T^{2}}$$

According to Onsager’s pioneering work [27,28], the dissipation function is introduced by analogy to the Rayleigh dissipation function,

(21) $$\Phi_{J}=\frac{q^{2}}{2l_{qq}}$$

with the phenomenological coefficient, *l_qq_*. Thus, the least dissipation of energy principle [27,28] leads to

(22) $$\delta \left[\sigma_{S}-\Phi_{J}\right]=0\Rightarrow q=l_{qq}\nabla \left(\frac{1}{T}\right)=-\frac{l_{qq}}{T^{2}}\nabla T$$

The phenomenological parameter, *l_qq_*, must be a constant [27,28]; thus, only when the thermal conductivity is inversely proportional to the square of temperature,

(23) $$k=\frac{l_{qq}}{T^{2}}$$

the constitutive equation, Equation (22), derived from the entropy production rate, can be in agreement with Fourier’s heat conduction law, although in fact, this requirement can rarely be satisfied in practice [18].

In contrast, the entransy dissipation rate can be employed to derive the principle of least action for heat conduction process. We have [25]

(24) $$\delta \left[\sigma_{g}-\Phi \right]=\delta \left[-q\cdot \nabla T-\frac{q^{2}}{2l_{qq}}\right]=0$$

whose variation with respect to **q** can give the constitutive relation between heat flux and temperature gradient,

(25) $$q=-l_{qq}\nabla T$$

In this way, Fourier’s heat conduction law is derived as $k=l_{qq}=\mathrm{constant}$, indicating that it is the entransy dissipation rate, rather than the entropy production rate, that is able to serve as the action for deriving the fundamental constitutive equation for the heat conduction process.

### 3.2. Heat Transfer Optimization on the Basis of Entransy Dissipation Rate

Optimization of a heat transfer process aimed at cooling or heating objects can be highly relevant to its action (i.e., the entransy dissipation rate) [15,16]. The extremum of total entransy dissipation rate can give the best heat transfer performance; therefore, the entransy dissipation extremum principle (EDEP) [15] was proposed for heat transfer optimization. In fact, the entropy production rate has been used to optimize heat transfer processes [29,30], but several counter-examples were identified, in which the minimization of the entropy production rate cannot correspond to the optimal performance of heat transfer [16,20]. The EDEP was firstly applied to optimizing heat conduction problems under given constraints [31,32,33]. In our previously published paper [25], a 1D volume-point problem was studied to illustrate the optimization results based on the entransy dissipation rate and the entropy production rate, respectively. The extremum of the total entransy dissipation rate can give the linear thickness distribution that leads to the minimum average temperature rise; however, the minimization of the total entropy production rate cannot lead to any solution for the optimal distribution. In addition, the principle can also be extended to handle thermal convection optimization problems [16]; this is because the convective heat transfer process is actually heat conduction with heat sources [34]. As for some complicated problems, such as heat exchanger networks [35,36,37], the entransy theory can be employed to simplify the constraints, and thus facilitate the optimization process. By introducing entransy dissipation-based thermal resistance [16],

(26) $$R_{h}=\frac{\sigma_{G}}{Q^{2}}$$

in which *Q* is the total heat transfer flow through the boundaries. A heat exchanger network can be converted to a thermal resistance network, and then the entransy balance condition can be used in the Lagrange variational method for optimization. 

## 4. Conclusions

(1) Lyapunov functions in the modern theory of stability can be used to characterize the irreversibility (that is, the one-sidedness of time) and the entropy production rate can usually serve as Lyapunov functions to measure the irreversibility of various transport processes.
(2) For heat conduction processes, a rigorous proof was given to demonstrate that the entransy and its dissipation rate can also serve as Lyapunov functions to reflect their irreversibility. These results indicate that the Lyapunov function is not unique for a specific heat conduction problem without the conversion between heat and work.
(3) The entransy dissipation rate is the action of heat conduction, which is unique and can be used to derive Fourier’s heat conduction law and to optimize heat transfer processes.
(4) Entropy, but not entransy, is the Lyapunov function for the isolated system from a non-equilibrium state to the equilibrium state, or the non-equilibrium system from an unsteady state to the steady state with the conversion between heat and work.
